# An antisite defect mechanism for room temperature ferroelectricity in orthoferrites

**DOI:** 10.1038/s41467-021-24592-w

**Published:** 2021-07-14

**Authors:** Shuai Ning, Abinash Kumar, Konstantin Klyukin, Eunsoo Cho, Jong Heon Kim, Tingyu Su, Hyun-Suk Kim, James M. LeBeau, Bilge Yildiz, Caroline A. Ross

**Affiliations:** 1 Department of Materials Science and Engineering, Massachusetts Institute of Technology, Cambridge, MA USA; 2 School of Materials Science and Engineering, National Institute for Advanced Materials, Nankai University, Tianjin, People’s Republic of China; 3 Department of Materials Science and Engineering, Chungnam National University, Daejeon, Korea; 4 Department of Nuclear Science and Engineering, Massachusetts Institute of Technology, Cambridge, MA USA

**Keywords:** Ferroelectrics and multiferroics, Surfaces, interfaces and thin films

## Abstract

Single-phase multiferroic materials that allow the coexistence of ferroelectric and magnetic ordering above room temperature are highly desirable, motivating an ongoing search for mechanisms for unconventional ferroelectricity in magnetic oxides. Here, we report an antisite defect mechanism for room temperature ferroelectricity in epitaxial thin films of yttrium orthoferrite, YFeO_3_, a perovskite-structured canted antiferromagnet. A combination of piezoresponse force microscopy, atomically resolved elemental mapping with aberration corrected scanning transmission electron microscopy and density functional theory calculations reveals that the presence of Y_Fe_ antisite defects facilitates a non-centrosymmetric distortion promoting ferroelectricity. This mechanism is predicted to work analogously for other rare earth orthoferrites, with a dependence of the polarization on the radius of the rare earth cation. Our work uncovers the distinctive role of antisite defects in providing a mechanism for ferroelectricity in a range of magnetic orthoferrites and further augments the functionality of this family of complex oxides for multiferroic applications.

## Abstract (web summary)

Ferroelectricity in orthoferrite perovskites has stimulated intense research, but the mechanism remains unclear. Here, the authors propose an antisite defect mechanism for introducing ferroelectricity in magnetically ordered YFeO3 and the family of rare earth orthoferrites.

## Introduction

Multiferroic materials combine ferroelectric and magnetic properties, and hold compelling interest for magnetoelectric and spintronic applications^1^. It has traditionally been a challenge for ferroelectricity and ferromagnetism to coexist in a single-phase material, including the perovskite-structured (ABO_3_) transition metal oxides^2^. While magnetoelectric coupling can be artificially realized by integrating ferroelectric and magnetic phases into composites or heterostructures^3^, single-phase multiferroic materials and mechanisms offer substantial fundamental and technological interest, and are highly desirable for practical applications.

One of the best-known single-phase multiferroics is BiFeO_3_ (ref. ^4^), which possesses large spontaneous polarization (~100 μC/cm^2^) driven by the stereochemical activity of lone-pair electrons of A-site Bi^3+^ ions and weak magnetization, due to the canted antiferromagnetic ordering of B-site Fe^3+^ ions^5,6^. Beyond this material, novel mechanisms for multiferroicity have been established by incorporating unconventional ferroelectricity into magnetic oxides^7^, such as the geometrically induced ferroelectricity due to polyhedral tilt and rotation in hexagonal manganites^8,9^ and ferrites^10,11^; electronically driven ferroelectricity due to charge ordering^12,13^; and magnetically induced ferroelectricity due to types of non-centrosymmetric spin ordering^14^. Strain engineering is also predicted to promote ferroelectricity in certain magnetic oxides, e.g., EuTiO_3_ (refs. ^15,16^) and SrMnO_3_ (refs. ^17,18^), by spin–lattice coupling.

Among such approaches, magnetically driven ferroelectricity is of great interest due to the inherent coupling expected between the ferroelectric and magnetic orderings. It was first reported in TbMnO_3_ that inversion symmetry breaking is caused by a cycloidal spiral spin structure at cryogenic temperatures^19^. Subsequently, ferroelectricity with a weak polarization (0.1 μC/cm^2^) was also observed in a family of rare-earth orthoferrites (RFeO_3_) with much simpler collinear spin structures, e.g., GdFeO_3_ (ref. ^20^) and DyFeO_3_ (ref. ^21^). The mechanism is attributed to the exchange-striction effect of R 4*f* and Fe 3*d* spins, and hence the ferroelectricity only occurs below the R 4*f* spin ordering temperature of a few Kelvin. In HoFeO_3_ (ref. ^22^) and SmFeO_3_ (ref. ^23^), ferroelectricity is reported to be present near or even above room temperature, but contradictory conclusions from theoretical^24^ and experimental^25,26^ work have obscured the mechanism, particularly in the case of room temperature ferroelectricity reported in YFeO_3_ (YFO)^27–30^.

YFO adopts a space group *Pbnm* with lattice parameters of *a*_o_ = 5.282 Å, *b*_o_ = 5.595 Å, and *c*_o_ = 7.605 Å (the subscript o denotes the orthorhombic notation). Such a centrosymmetric structure inhibits spontaneous polarization in principle. The unexpected ferroelectricity found in YFO was initially attributed to spin–orbit coupling^27^, i.e., the inverse Dzyaloshinskii–Moriya interaction (DMI), as proposed for SmFeO_3_ (ref. ^23^). However, it was shown that inverse DMI is unable to break the inversion symmetry in SmFeO_3_ (ref. ^24^), precluding this mechanism. The ferroelectricity also cannot stem from spin exchange interactions because of the empty 4*f* orbital of Y^3+^ ions. Moreover, the reported polarization of YFO varies dramatically in magnitude from bulk (<0.01 μC/cm^2^)^27^ to one thin film report (~10 μC/cm^2^)^28^, challenging the interpretations of the origin of its polarization.

In this work, we select YFO as a model orthoferrite to investigate the mechanism that yields a robust, sizeable, and switchable ferroelectric polarization above room temperature, while preserving its magnetization. We find that, contrary to the fact that the ferroelectricity generally deteriorates upon the presence of cationic off-stoichiometry in incipient ferroelectric CaTiO_3_ (ref. ^31^), and analogous to the findings that cationic off-stoichiometry yields a ferroelectric distortion in antiferroelectric PbZrO_3_ (ref. ^32^) or paraelectric SrTiO_3_ (refs. ^33,34^), the presence of Y–Fe antisite (Y_Fe_) defects in Y-rich YFO plays a crucial role in facilitating a non-centrosymmetric distortion, which promotes a spontaneous polarization, but preserves the magnetic order. The polarization persists over a range of film thicknesses, Y-rich compositions, and lattice strains, and is supported by both theoretical simulations and experimental characterization. Density functional theory (DFT) calculations demonstrate that such an antisite defect mechanism is also expected for other rare-earth orthoferrites, in which the polarization depends on the radius of the rare-earth cation. Overall, our results demonstrate a cationic antisite defect mechanism for ferroelectricity in the family of orthoferrites, offering a strategy for designing single-phase multiferroics.

## Results

### Unexpected ferroelectricity in magnetic YFO thin films

**Fig. 1:**
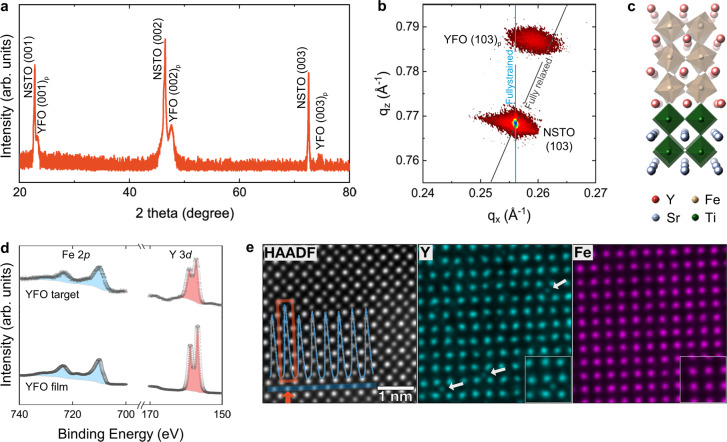
Structure and defect analysis of YFO thin films on NSTO substrate. **a**, **b** Structural characterization of the as-grown YFO/NSTO by high-resolution XRD (**a**) and asymmetric RSM (**b**). The subscript p denotes the pseudocubic unit cell. **c** Schematic of lattice structures of YFO and NSTO viewed along the orthorhombic [110] axis (pseudocubic [100] axis) of the YFeO_3_ unit cell. **d** High-resolution Fe 2*p* and Y 3*d* core level spectra of the stoichiometric YFO target and the as-prepared YFO film. **e** HAADF STEM image and denoised atomic resolution STEM EDS elemental mapping. The intensity profile superposed in the HAADF STEM image is taken along the blue line. Fe–O atom columns rich in Y_Fe_ defects show increased intensity in HAADF (red arrow) and a signal in the Y map (white arrows).

YFO thin films (~30 nm thick, Supplementary Note 1) were grown on 001-oriented Nb-doped SrTiO_3_ (NSTO; *a* = 3.905 Å) substrates by pulsed laser deposition (PLD, see “Methods”), using a stoichiometric YFO ceramic target. High-resolution X-ray diffraction (XRD, Fig. 1a) reveals that the YFO film exhibits an epitaxial perovskite structure, while the asymmetric (013) reciprocal space mapping (RSM, Fig. 1b) shows it is not fully strained to the substrate. The broadening of the RSM peak suggests that imperfections, such as mosaicity exist. With the YFO lattice described using a pseudocubic unit cell (Fig. 1c), the in-plane (*a*_p_, the subscript p denotes the pseudocubic notation) and out-of-plane (*c*_p_) lattice parameters of the YFO film are 3.862 and 3.813 Å, respectively, both of which are larger than the bulk values (*a*_p, bulk_ = $\frac{1}{2}\scriptstyle\sqrt{{{a}_{{\rm{o}}}}^{2}+{{b}_{{\rm{o}}}}^{2}}\,$ = 3.847 Å, *c*_p, bulk_ = *c*_o_/2 = 3.803 Å), i.e., the unit cell volume of the thin film is larger by +1.04% compared to bulk.

The chemical composition analyzed by high-resolution X-ray photoelectron spectroscopy (XPS) reveals that the as-prepared YFO film possesses a Y-rich composition, which is surprisingly off-stoichiometric compared to the target (Fig. 1d). XPS yielded a Y/Fe ratio of *α* = 1.19 ± 0.04 for five samples prepared during several PLD runs under the same deposition conditions (“Methods”, Supplementary Note 1, and Supplementary Table 1). Quantitative energy-dispersive spectroscopy (EDS) elemental analysis using a Y:Fe standard was conducted on a cross-section of one sample of YFO/NSTO with Y/Fe ratio *α* = 1.19, using scanning transmission electron microscopy (STEM). EDS reveals a uniform composition within the sample with Y/Fe ratio of 1.21 ± 0.12, further corroborating the Y-rich nature of the films (Supplementary Note 1). This cationic off-stoichiometry can arise from differences in ablation or scattering rate of Y and Fe during the PLD process^35^.

Atomically resolved STEM EDS (Fig. 1e) collected on the YFO/NSTO sample with *α* = 1.19 reveals the presence of Y ions at Fe sites, i.e., Y_Fe_ defects. Based on the composition and the lamella thickness (11 nm), two to three antisite defects on average are expected in each Fe–O atom column. While the STEM EDS does not have the sensitivity to resolve such small numbers of antisite defects, significant EDS intensity in the Y elemental map is observed for those Fe atom columns containing a statistically larger number of Y_Fe_ defects. Further analysis of the simultaneously acquired, atomic number (*Z*)-sensitive high-angle annular dark-field (HAADF) STEM reveals an increase in atom column intensity of 10–20% at these locations, confirming the presence of Y (*Z* = 39) in the Fe (*Z* = 26) sites. Soft X-ray absorption spectroscopy (XAS) analysis at both the Fe *L*-edge and O *K*-edge shows that Fe primarily adopts a +3 valence state (Supplementary Fig. 2). Given that both Fe and Y exist as trivalent ions, deviation from the ideal cation stoichiometry *α* = 1 can be accommodated without changes in the oxygen content.

**Fig. 2:**
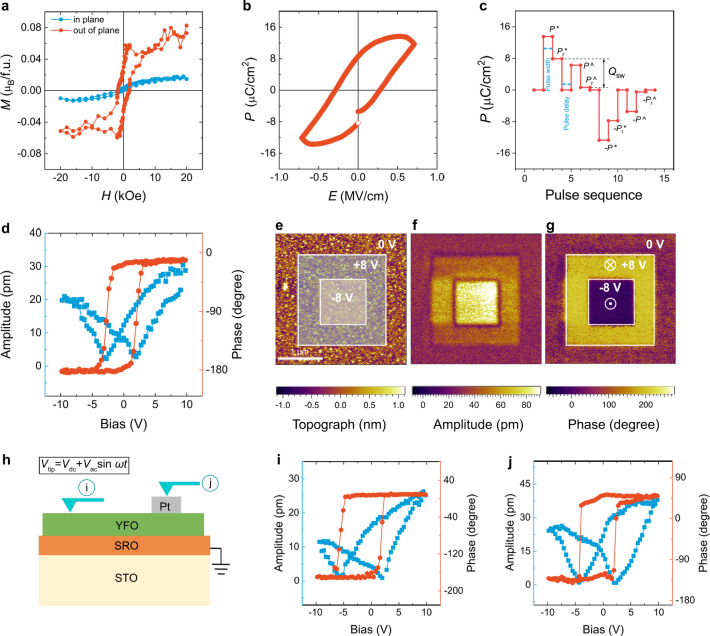
Magnetic and ferroelectric properties of YFO thin films. **a** *M–H* curves measured at 5 K. **b** Macroscopic *P–E* loop measured at room temperature with a frequency of 50 Hz. **c** PUND measurements with pulse width of 1 ms and pulse delay of 100 ms. *Q*_SW_ is the switched charge density. **d** Local SS-PFM amplitude curve and phase loop. **e**–**g** Box-in-box writing experiments carried out as indicated in the topographic image (**e**). The vertical PFM amplitude (**f**) and vertical PFM phase (**g**) images are collected subsequently. **h** Cross-sectional schematic of SS-PFM measurements with the cantilever loaded on the surface of YFO film or on the Pt electrode. **i**, **j** Local SS-PFM amplitude curves and phase loops collected with the cantilever on the YFO film (**i**) and on the top of Pt electrode (**j**), respectively.

Bulk YFO is a perovskite-structured antiferromagnet below its Néel temperature (*T*_N_) of 645 K with a Bertaut notation spin structure of G_*x*_A_*y*_F_*z*_, possessing a small net magnetization parallel to the *c*_o_-axis as a result of the canting of the Fe^3+^ moments^36^. Magnetometry analysis of a YFO/NSTO sample with *α* = 1.19 (Fig. 2a) shows a hysteretic response along the out-of-plane direction with a saturation magnetization (*M*_s_) of ~0.056 μ_B_/f.u., comparable with that of bulk^37^, and indicating that the out-of-plane orientation is the orthorhombic *c*_o_-axis. In contrast, the in-plane loop is not saturated at a field of 20 kOe. Anisotropic hysteresis loops are also reported in bulk single-crystal YFO^38^.

Contrary to the nonferroelectric nature of bulk YFO, a sizeable ferroelectric polarization is observed at room temperature in the YFO/NSTO (*α* = 1.19), as revealed by the polarization-electric field (*P–E*) hysteresis loop (Fig. 2b). Positive-up-negative-down (PUND) pulse measurements are shown in Fig. 2c. The total remanent polarization (*P*_r_*) of 9.5 μC/cm^2^ after the first pulse includes both switched charge density (*Q*_SW_) and leakage current, and the remanent polarization (*P*_r_^) of 2.3 μC/cm^2^ after the second positive pulse originates mainly from leakage current. The switched charge density, *Q*_SW_ = *P*_r_* − *P*_r_^ = 7.2 μC/cm^2^ indicates an intrinsic and switchable remanent polarization. The polarization observed here is orders of magnitude stronger than the values reported previously in bulk RFeO_3_, R = Gd^20^, Dy^21^, Ho^22^, and Sm^23^, and comparable with a measurement reported by Shang et al. in thin film YFO^28^.

The ferroelectric nature is confirmed by switching spectroscopy piezoresponse force microscopy (SS-PFM). The vertical PFM phase hysteresis loop and the “butterfly-shape” amplitude curve (Fig. 2d) and the piezoresponse hysteresis (Supplementary Fig. 3d) demonstrate clear reversal. This switching behavior does not qualitatively depend on the film thickness within the range of 10–100 nm, indicating that is it not greatly affected by strain relaxation, and persists at elevated temperature up to at least 150 °C, the limit of the instrument (Supplementary Fig. 4). Box-in-box writing with DC voltages of ±8 V as shown in Fig. 2e results in 180° phase contrast in the vertical PFM phase image (Fig. 2g), indicating a complete switching of polarization between upward and downward orientations. Scanning Kelvin probe force microscopy (SKPFM) was performed to exclude the possibility that the PFM signal originates from nonferroelectric mechanisms (Supplementary Note 2).

YFO was also grown on SrRuO_3_ (SRO, ~10 nm thick)-buffered STO substrates. YFO/SRO/STO adopts a similar crystal structure and Y-rich stoichiometry as YFO/NSTO. Almost the same ferroelectric switching behaviors are found whether the cantilever in PFM is placed directly on the YFO film surface or on a Pt top electrode (Fig. 2h–j), further excluding surface and interface artifacts. We also prepared YFO films on different substrates with conductive layers (see “Methods”), including SRO-buffered DyScO_3_ (DSO, *a*_p_ = 3.944 Å), La_0.67_Sr_0.33_MnO_3_ (LSMO)-buffered (LaAlO_3_)_0.3_–(SrAl_0.5_Ta_0.5_O_3_)_0.7_ (LSAT, *α*_p_ = 3.868 Å), and LSMO-buffered LaAlO_3_ (LAO, *α*_p_ = 3.788 Å). Again, Y-rich stoichiometry and ferroelectric switching are consistently observed across these samples (Supplementary Fig. 6), but a small dependence of the remanent piezoresponse on the substrate lattice parameter (Supplementary Table 2) suggests that the epitaxial strain plays a minor role in the piezoresponse.

### Dependence of ferroelectricity on the cation stoichiometry

**Fig. 3:**
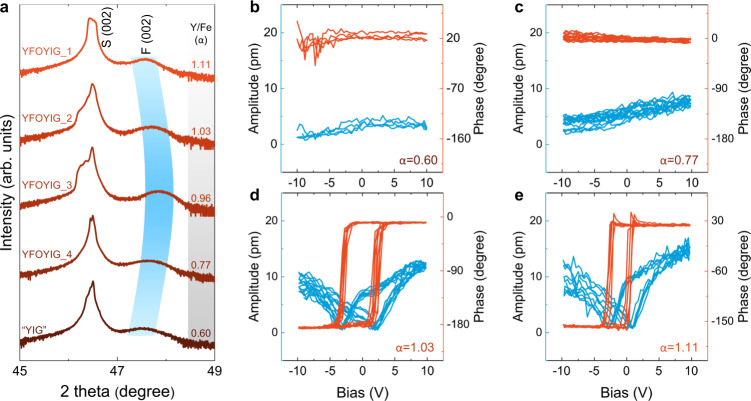
Dependence of structure and ferroelectric switching on the Y/Fe ratio. **a** High-resolution XRD pattern of the 002 family of peaks of the film deposited from the YIG target and co-deposited Y_*α*_FeO_1.5(*α* + 1)_ films grown on NSTO substrates. S and F refer to the peaks of substrate and film. The Y/Fe ratio, i.e., *α*, determined by high-resolution XPS analysis is labeled correspondingly. **b**–**e** Local SS-PFM amplitude curves and phase loops of “YIG” films (*α* = 0.60) (**b**) and co-deposited Y_*α*_FeO_1.5(*α* + 1)_ thin films with *α* = 0.77 (**c**), 1.03 (**d**), and 1.11 (**e**), respectively.

The effects of cation stoichiometry on the ferroelectricity were first evaluated by growing a sample using a Y_3_Fe_5_O_12_ (yttrium iron garnet, YIG) target. High-resolution XPS suggests that the as-prepared “YIG”-composition thin film on NSTO substrate shows almost the same Y/Fe ratio as the target, i.e., *α* = 0.6, while the STEM EDS analysis indicates compositional heterogeneity with regions having relative excess of Y or Fe, with respect to the average *α* = 0.6 (Supplementary Note 5). The film exhibits a perovskite structure (Fig. 3a), which is qualitatively different from the garnet structure of bulk YIG. A series of Y_*α*_FeO_1.5(*α* + 1)_ (0.60 < *α* < 1.19) thin films were then prepared on NSTO by codeposition from the YFO and YIG targets. All of the films adopt the perovskite structure (Supplementary Fig. 7b).

The ferroelectric behavior depends strongly on the Y/Fe ratio as revealed by SS-PFM. For the Y-deficient regime *α* < 1 no switching is found (Fig. 3b, c), while for the Y-rich regime, i.e., *α* = 1.03 (Fig. 3d), 1.11 (Fig. 3e), and 1.19 (Fig. 2d), consistent ferroelectric switching is observed. The out-of-plane lattice dimension gradually expands (Fig. 3a) as *α* becomes either greater or smaller than 1, as a consequence of cation defects. Comparing samples with *α* = 1.11 and *α* = 0.60, the lattice parameters and unit cell volume are almost the same, but the ferroelectric response is markedly different, pointing to composition as the key factor. Moreover, as *α* decreases, the remanent piezoresponse gradually weakens (Supplementary Fig. 7i). We therefore conclude that the Y/Fe stoichiometry plays the dominant role in the room temperature ferroelectricity of epitaxial YFO thin films.

### Mechanism of ferroelectricity induced by Y_Fe_ defects

To explain the physical basis of these experimental results, first-principles DFT simulations were employed to calculate the electronic structure and atomic positions of Y-rich YFO (see “Methods”). The formation energy of various types of point defects in the perovskite YFO lattice evaluated with appropriate chemical potentials for each element suggests that the Y_Fe_ (or Fe_Y_) antisite defects have much lower formation energies than cation vacancies (Supplementary Note 6), which is consistent with the result of first-principles calculations in YIG^39^ and the presence of Y_Fe_ in our as-prepared Y-rich YFO film. The Y_Fe_ defects are stabilized by tensile in-plane strain, and are unlikely to migrate upon the application of moderate external voltages because of a high migration barrier of 3.1 eV (Supplementary Note 6).

**Fig. 4:**
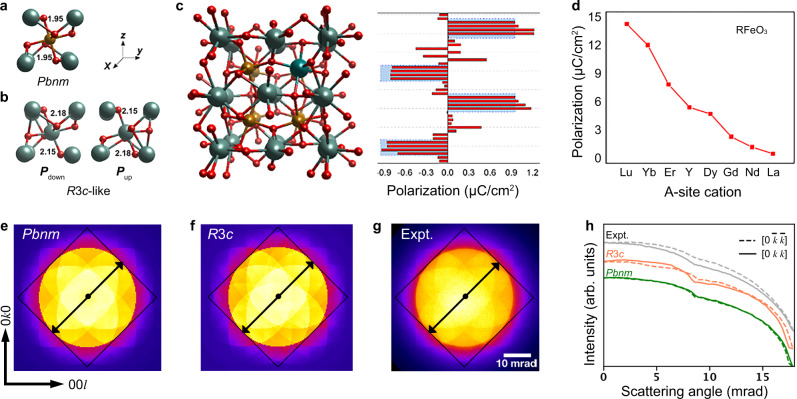
Crystallographic origin of ferroelectricity in Y-rich YFO. **a**, **b** Schematics of centrosymmetric *Pbnm* (**a**) and non-centrosymmetric *R3c*-like (**b**) structures. **c** Crystal structure and layer-resolved polarization for Y-rich YFO. Red columns indicate the atomic contribution to the ferroelectric polarization in Y-rich YFO calculated using the Born charge approximation, while blue columns show layer-resolved contributions to the ferroelectric polarization in stoichiometric YFO. **d** Ferroelectric polarization for various orthoferrites RFeO_3_ that are assumed to be epitaxially grown on SrTiO_3_ for all calculations with R/Fe = 1.28. **e**–**g** PACBED patterns including the simulated patterns, using the DFT relaxed centrosymmetric *Pbnm* (**e**) and non-centrosymmetric *R3c* (**f**) and the experimental result measured from the Y-rich YFO (*α* = 1.19) thin film sample (**g**). **h** Intensity profiles integrated across the pattern diagonals from PACBED patterns along the black arrows shown in **e**–**g**.

The symmetry calculation suggests the origin of the ferroelectricity lies in the structural distortion around the Y_Fe_ sites, where the *Pbnm* structure (Fig. 4a) is locally distorted into a region with *R*3*c*-like symmetry (Fig. 4b). In YFO, the changes in bonding of oxygen atoms adjacent to Y_Fe_ break the inversion symmetry, generating a net local dipole moment (Fig. 4b), which further polarizes the surrounding stoichiometric regions, producing a spontaneous polarization. The layer-resolved polarization for Y-rich YFO, calculated using the Born charge approximation, is given in Fig. 4c. Both Y_Fe_ defects and epitaxial strain promote the formation of the *R*3*c* regions within the *Pbnm* YFO structure (Supplementary Note 7).

Position-averaged convergent beam electron diffraction (PACBED), which is sensitive to inversion center symmetry breaking^40^, confirms the crystallographic origins of ferroelectricity. Compared to the simulated patterns using the DFT relaxed structures of both centrosymmetric *Pbnm* (Fig. 4e) and non-centrosymmetric *R*3*c* (Fig. 4f), the experimental pattern (Fig. 4g) measured from the Y-rich YFO (*α* = 1.19) film exhibits an asymmetric feature, i.e., a mismatch of intensity profiles between [0 *k k*] and $[0\;\bar{k}\;\bar{k}]$ directions (Fig. 4h), which is consistent with the simulated non-centrosymmetric *R*3*c*, but different from the centrosymmetric *Pbnm* pattern where the intensity profile along [0 *k k*] coincides exactly with that along $\left[0\;\bar{k}\;\bar{k}\right]$. These results support the non-centrosymmetric structure predicted by our DFT calculations and provide evidence that the Y_Fe_ defects induce inversion symmetry breaking that can support ferroelectricity.

The DFT calculations predict a switchable out-of-plane polarization of 7.2 μC/cm^2^ for YFO with *α* = 1.28 (Supplementary Note 7), in reasonable agreement with our experimental results. The calculations also indicate that ordering of Y_Fe_ antisites has little effect on the spontaneous polarization (Supplementary Fig. 10). *R*3*c* structures in many other RFeO_3_ orthoferrites with magnetic and nonmagnetic A-site ions (R = Lu, Yb, Er, Y, Dy, Gd, Nb, and La) were also modeled. The R_Fe_ antisite defects favor ferroelectric behavior (Fig. 4d) across the series, suggesting the general nature of this mechanism to yield multiferroicity in orthoferrites. In the *R*3*c* structure the spontaneous polarization falls monotonically with an increase of the ionic radius of the rare-earth cation. DFT further shows that the antiferromagnetic order in the YFO is robust in the presence of Y_Fe_ (Supplementary Note 7).

To summarize, our study reveals a mechanism for ferroelectricity based on antisite defects in the perovskite-structured canted antiferromagnet YFO with excess Y. The Y_Fe_ defects play a dominant role in promoting a non-centrosymmetric structural distortion that yields a switchable polarization, which is robust above room temperature, and present in films grown with a range of strain states and film thicknesses. The antisite defects are stable and occur without changes in oxygen content because the Y and Fe cations are isovalent.

According to first-principles calculations, the relationship between cation off-stoichiometry and ferroelectricity is expected to occur generally within the family of orthoferrites, with magnitude depending on the specific A-site cation. Given the rich magnetic phase diagram particularly for those RFeO_3_, where R has partially filled *f* electrons, it is of substantial interest and significance to investigate the influence of antisite defects on the magnetic properties, and the interactions between ferroelectric and magnetic orderings. Our work therefore opens a route for promoting ferroelectricity and multiferroicity in single-phase orthoferrites.

## Methods

### Thin film preparation

The YFO thin films were prepared by PLD using a KrF excimer laser (*λ* = 248 nm) with 1.3 J/cm^2^ fluence and 10 Hz repetition rate to ablate a ceramic YFO target. The setpoint temperature of the substrate holder was 900 °C and the substrate itself was ~100°C below this. The oxygen partial pressure, *p*(O_2_), was 10 mTorr. After growth, the films were cooled down to room temperature in the same *p*(O_2_) at a rate of 20 °C/min. For those grown on nonconductive substrates, a 10-nm-thick SRO layer was grown on STO or DSO at 850 °C substrate holder temperature under *p*(O_2_) = 5 mTorr, and a 15-nm-thick LSMO layer was grown on LSAT or LAO at 800 °C substrate holder temperature under *p*(O_2_) = 10 mTorr. The “YIG” thin films were grown on NSTO substrates at the same conditions by ablating a ceramic YIG target. A series of Y_*α*_FeO_1.5(*α* + 1)_ thin films were prepared by codeposition using YFO and YIG targets.

### Composition and structural characterization

The chemical composition is analyzed by using a Thermo Scientific K-Alpha+ XPS system with Al *Kα* (1486.6 eV) as the X-ray source. Before collecting the data, the sample surface was cleaned with a cluster Ar-ion beam for 30 s. For quantitative analysis of the Y/Fe ratio of YFO films, the stoichiometric YFO target whose Y/Fe ratio is 1:1 was measured, and the ratio of the integrated areas of Y 3*d* and Fe 2*p* core level spectra was taken as a reference. XAS measurements at Fe *L*-edges and O *K*-edges were performed in total electron yield (TEY) modes, using the beamline 4-ID-C of the Advanced Photon Source at Argonne National Laboratory. The temperature was set to 200 K to obtain a good signal to noise ratio. Reference scans of elemental Fe measured simultaneously indicate negligible energy shift throughout the experiments. The film thickness was analyzed by X-ray reflectivity, and the crystalline structure was characterized by both high-resolution XRD and RSM, using a Rigaku SmartLab high-resolution diffractometer with Cu *Kα*_1_ radiation (*λ* = 1.5406 Å) as X-ray source and an incident beam Ge-(220) double-bounce monochromator.

### Transmission electron microscopy

Cross-sectional samples of thin film YFO were prepared for electron microscopy by mechanical wedge polishing and final thinning, using cryogenic Ar-ion milling. STEM analysis was performed with a probe corrected Thermo Fisher Scientific Titan G3 60-300 kV operated at 200 kV with a probe convergence semiangle of 18 mrad. A collection semiangle range of 63–200 mrad was used for STEM imaging. Atomic resolution EDS elemental maps were collected with a Thermo Fisher Scientific Super-X EDS detector, and Y and Fe elemental maps were denoised using nonlocal principal component analysis, and gaussian blurring using an open-source Matlab script^41^. The Y/Fe ratio was quantified using the stoichiometric YFO target as reference. PACBED patterns were simulated with a custom Python-based STEM simulation, using the multislice approach^42^.

### Magnetic and ferroelectric properties measurements

Magnetic properties were analyzed using a Quantum Design MPMS-3 SQUID magnetometer and a Digital Measurement System 7035B vibrating sample magnetometer. In the SQUID magnetometer out-of-plane *M–H* measurements were performed on a 2 mm × 2 mm YFO film sample mounted on a semicylindrical quartz holder, while in-plane measurements were performed on a 4 mm × 4 mm sample, yielding a higher signal to noise ratio. A Precision Premier II Ferroelectric Tester was used to perform the *P–E* loops and PUND measurements with a home-made probe station. SS-PFM, PFM, and SKPFM measurements were performed on a commercial atomic force microscope (Cypher, Asylum Research) under dual-frequency resonant tracking modes with Pt-coated Si conductive probes (MikroMasch, HQ:NSC18/Pt).

### Density functional theory calculation

First-principles calculations using DFT were first performed to evaluate the formation energy of various point defects in YFO, using the projector augmented wave method as implemented in Vienna Ab initio Simulations Package^43,44^. The plane wave energy cutoff of 500 eV and Monkhorst-Pack *k*-point sampling were used. The YFO unit cell is calculated using a 4 × 4 × 4 *k*-point mesh with generalized gradient approximation for which the Perdew–Burke–Ernzerhof functional was used^45^. Defect calculations were performed on 80 atoms in a 2 × 2 × 1 supercell (or a 3 × 4 × 4 supercell for Supplementary Fig. 9) with a Monkhorst-Pack 2 × 2 × 2 mesh. The total energies and forces were converged to <10^−6^ eV and 5 meV/Å, respectively. Ferroelectric properties were calculated using the Berry-phase approach^46^. A pseudocubic ($\sqrt{2}$*a*_o_ × $\sqrt{2}$*b*_o_ × *c*_o_) *Pbnm* supercell with a single Y_Fe_ antisite defect, and fixed in-plane lattice parameters was used to simulate Y/Fe non-stoichiometry and epitaxial growth on a SrTiO_3_ substrate. Born effective charges approximation was used to derive atomic-resolved contributions to ferroelectric polarization. To estimate polarization switching barriers, we calculated the migration energy profile along the minimum energy path between two polarization states, using the climbing image nudged elastic band method^47^. The choice of exchange-correlation functional is discussed in Supplementary Note 7.

## Supplementary information

Supplementary Information

## Data Availability

The data that support the findings of this study are available from the corresponding author on reasonable request.
