# Supplementary material for: An antisite defect mechanism for room temperature ferroelectricity in orthoferrites
Source: Nat Commun. 2021 Jul 14;12:4298. doi: 10.1038/s41467-021-24592-w (PMC8280199; doi:10.1038/s41467-021-24592-w)
Supplement: Supplementary file 1 — Supplementary Information [file 41467_2021_24592_MOESM1_ESM.pdf]

Supplementary Information

An antisite defect mechanism for room temperature ferroelectricity in orthoferrites

Shuai Ning[1,2*], Abinash Kumar[1], Konstantin Klyukin[1], Eunsoo Cho[1], Jong Heon Kim[3], Tingyu Su[1], Hyun-Suk Kim[3], James M. LeBeau[1], Bilge Yildiz[1,4] and Caroline A. Ross[1*]

[1]Department of Materials Science and Engineering, Massachusetts Institute of Technology, Cambridge, Massachusetts 02139, USA.

[2]School of Materials Science and Engineering, National Institute for Advanced Materials, Nankai University, Tianjin 300350, P. R. China.

[3]Department of Materials Science and Engineering, Chungnam National University, Daejeon 305-764, Korea

[4]Department of Nuclear Science and Engineering, Massachusetts Institute of Technology, Cambridge, Massachusetts 02139, USA.

[*]E-mail: S. N.: sning@nankai.edu.cn; C. A. R.: caross@mit.edu

Contents:

Notes 1-7

Tables 1-4

Figures 1-10

References 1-12

Supplementary Note 1. Microstructure, composition and chemical state analysis of YFO/NSTO.

Composition of Films. We conducted composition analysis by high-resolution XPS on multiple YFO films (#1-5) grown on NSTO substrates under the same PLD conditions. For the five different samples, the average Y/Fe ratio is 1.19 ± 0.04 (Supplementary Table 1), indicating the Y-rich nature of these as-prepared YFO films.

XPS is known to be surface-sensitive. To confirm the composition measurements were representative of the interior of the film, we carried out STEM imaging (Supplementary Fig. 1a, b) on the YFO thin film #1, whose Y/Fe ratio is 1.19 by XPS, in cross section along the pseudocubic [100]. Bright field STEM images reveal that the YFO thin film shows mosaicity with slight deviations of the crystal orientations. STEM EDS was used to map the elemental distribution across the thin film #1 (Supplementary Fig. 1c, d). The Cliff-Lorimer factor (*k*-factor) used for EDS quantification was experimentally determined to be 1.384 ± 0.136 using the stoichiometric PLD target as a reference with Y:Fe = 1:1. From EDS of film #1, the cation atomic fractions of Y and Fe are 54.75 ± 3.83 at% Y, 45.25 ± 3.16 at% Fe, respectively, yielding a Y: Fe ratio of 1.21 ± 0.12. In combination with the XPS measurement, the quantitative EDS analysis further supports the conclusion that this film is Y-rich.The YFO film #1 also exhibits a uniform composition with Y:Fe composition standard deviation 0.7 at % measured at several different locations within the sample.

Error analysis of composition determination. For the EDS compositional analysis, we consider two sources of error: net counts and the k-factor error. The integrated net signal counts dictate the precision of the measurement, and was in the range of millions of net counts for each

element. A 1% precision within a 99% confidence interval requires at least 0.1 million net count/intensity, which we have far surpassed in our experiment, with 2-3 million net count for each element in all the samples. If we consider only these counting statistics, the precision of the composition would be 1.21±0.0049.

The second error arises from estimating the k-factors used to determine the composition. The k-factors provided by the Thermo Fisher Scientific EDS analysis software take into account X-ray yields, cross sections for the corresponding elements, and the calibrated response of the EDS detector. These calculated k-factors can introduce relative error up to ~15-20%. However, using the YFO target as a reference reduces this error by a factor of about 2, leading to the Y: Fe ratio of 1.21 ± 0.12 stated above.

Oxidation state determination. The oxidation states are analyzed by XAS at both the Fe *L*-edge and O *K*-edge (Supplementary Fig. 2). The Fe *L*-edge XAS spectra results from Fe 2p to Fe 3d transitions. Specifically, peak I (707.6 eV) arises from transitions to non-bonding Fe 3d t_{2g} orbitals, while Peak II (709.3 eV) is ascribed to transitions to anti-bonding Fe 3d e_g states. This L_3-edge feature confirms that Fe adopts the +3 valence state as seen in stoichiometric bulk orthoferrites [1].

Further support is provided by the O *K*-edge XAS spectra, which corresponds to the excitation of O 1s electrons to anti-bonding states that originate from O 2p orbitals interacting with orbitals on the coordinating atoms, in which A, A′, C, and C′ are assigned to transitions O 2p to Fe 3d t_{2g}, O 2p to Fe 3d e_g, and O 2p to Fe 4p/4s states respectively, while B and B′ are related to the interactions between O 2p and Y 4d orbitals. In particular, the crystal field splitting of 1.6 eV, *i.e.* the difference between A and A′, is close to the value of 1.7 eV, the difference between Peak I and

II in the Fe L_3-edge spectra, are in good agreement with a previous study on bulk $YFeO_3$ compounds [1], indicating negligible oxygen deficiency in the YFO films.

Supplementary Note 2. Characterization of ferroelectric property of YFO/NSTO.

The resistivity of a 45 nm thick YFO (Y/Fe = 1.18) film on an insulating substrate (STO) was measured using a probe station to obtain an I-V curve up to 1 V. Averaging over multiple measurements gave a resistance of $2x10^{12}$ Ω equivalent to a resistivity of ~$2x10^{7}$ Ω cm. This is within the range of reports of ferroelectric $BiFeO_3$ (BFO) films, with resistivity $1x10^{7}$ Ω cm to ~$5x10^{8}$ Ω cm (estimated from current at 1 V)[2,3]. The resistivity suggests that leakage is present but the PUND data and the slight concavity of the P-E loop indicate a ferroelectric response with a switched charge density (Fig. 2c).

Supplementary Fig. 3a shows the piezoresponse hysteresis, PR(V), loop that is calculated according to PR(V) = Amp(V) • cos [ϕ(V)] with the amplitude, Amp(V), and phase, ϕ(V), shown in Fig. 2d, which clearly demonstrates the ferroelectric characteristic of the YFO film on NSTO substrate.

Supplementary Fig. 3b, c show the lateral PFM signals collected simultaneously with the vertical ones shown in Fig. 2f, g. Unlike the vertical PFM phase image (Fig. 2g), no clear 180° phase contrast was seen in the lateral PFM phase image (Supplementary Fig. 3c) between the regions poled by opposite voltages, indicating there is negligible in-plane component of the polarization. The contrast in both lateral PFM amplitude (Supplementary Fig. 3b) and lateral PFM phase (Supplementary Fig. 3c) images is only observed at the boundaries between regions written by opposite voltages can be a result of surface charge trapping.

Scanning kelvin probe force microscopy (SKPFM) was performed to rule out possible non-ferroelectric mechanisms, *e.g.* surface-trapped charges, that may contribute to signals in PFM

characterization. After poling, an apparent surface potential contrast is observed (Supplementary Fig. 3d): the positively (negatively) poled area shows higher (lower) surface potential. After multiple contact-mode scans over the same area with the tip grounded, legible surface potential contrast remains (Supplementary Fig. 3e) except for a slight decrease in the potential difference due to the removal of excessive screening charges on the surface. The remaining surface potential contrast arises from the difference in the surface charges that assist in stabilizing the polarization with opposite orientations poled by negative and positive biases.

Supplementary Note 3. Thickness and temperature dependence of SS-PFM.

YFO thin films with thickness ranging from 10 to 100 nm on NSTO substrates were prepared under the same PLD conditions. SS-PFM measurements reveal that ferroelectric behavior is always present irrespective of the film thickness, as seen in Supplementary Fig. 4a, b. Note that the 10-nm-thick film will break down under bias >7 V.

The measurements were also performed at elevated temperatures for the 30-nm-thick YFO film, *i.e.* 100 ˚C and 150 °C (Supplementary Fig. 4c, d). The results suggest that the ferroelectric switching still persists at temperature up to 150 °C, the limit of the instrument. Compared to that measured at room temperature (Fig. 2d), we notice that coercive voltage increases significantly at elevated temperature. This is due to the fact that the ferroelectric polarization usually becomes more active as temperature increases. Higher voltages are required to fully switch and stabilize the polarization [4].

Supplementary Note 4. YFO films grown on different substrates.

High-resolution XPS analysis shows that the YFO film grown on SRO/STO is also Y-rich with a Y/Fe ratio of 1.11. High-resolution XRD (Supplementary Fig. 5a) and (013) RSM

(Supplementary Fig. 5b) indicate the YFO film also exhibits a slight strain relaxation, similar to that grown on NSTO substrate and attributed to imperfections like mosaicity. We deposited Pt by sputtering as top electrodes and performed the SS-PFM measurements with the cantilever loaded directly on the surface of YFO thin film and on the Pt top electrodes (Fig. 2h). Almost the same amplitude curves and phase loops (Fig. 2i, j) and piezoresponse hysteresis loops (Supplementary Fig. 5c, d) were obtained. Box-in-box writing and rewriting were also performed on YFO/SRO/STO. Vertical PFM amplitude and phase images (Supplementary Fig. 5e, f) clearly reveal domain switching.

We also prepared YFO thin films under the same conditions on other different substrates with suitable conductive layers. High-resolution XPS was conducted to analyze the chemical composition, and the Y/Fe ratio is 1.12, 1.19 and 1.17 for the YFO films grown on SRO/DSO, LSMO/LSAT and LSMO/LAO (Supplementary Table 2), respectively.

Structural characterization shows that the 10~15 nm-thick conductive layers are coherently strained to the substrates in all cases, allowing us to evaluate the effects of epitaxial strain (ranging from +2.5% to −1.5%) on the ferroelectricity of the YFO films. With a decrease of the substrate lattice parameter, the out-of-plane lattice parameter (c_p) of YFO thin films increases indicated by the left shift of the 002 peak (Supplementary Fig. 6a). For the YFO film grown on SRO/DSO (Supplementary Fig. 6b), a significant relaxation is present, which is similar to that grown on SRO/STO (Supplementary Fig. 5b). For the YFO grown on LSMO/LSAT (Supplementary Fig. 6c), the film peak is largely overlapped with the substrate peak due to the similar lattice parameters but a slight relaxation can be noticed. For the YFO grown on LSMO/LAO, the film peak is overlapped with the Kiessig fringes of the LSMO layer (Supplementary Fig. 6a, d). While the out-of-plane lattice parameter can be extracted from the high-resolution XRD, the identification of the

YFO peak in RSM and hence the interpretation of in-plane lattice parameter is challenging, but if we assume the YFO is lattice matched to the substrate, the unit cell volume is smaller than bulk. Our DFT calculations indeed indicate a lattice volume shrinkage of -0.19% for an in-plane compressive strain of -1.0%, but a lattice expansion of +3.2% for an in-plane tensile strain of +1.0%. This trend has also been reported in other perovskite oxides, *e.g.* $SrRuO_3$ [5] and $LaCoO_3$ [6]. The LAO substrate with LSMO layer in our work provides a large compressive strain for YFO. Therefore, it is reasonable that the unit cell volume is smaller than that of bulk.

We summarize the lattice parameters of the YFO thin films grown on different substrates in Supplementary Table 2, with those of YFO/SRO/STO included as well. With strain varying from tensile to compressive, the c/a ratio changes from smaller to greater than 1. In addition, the pseudocubic unit cell volume shows a shrinkage for the compressive strain regime. Despite these differences in the strain states, room temperature ferroelectric switching is obtained consistently (Supplementary Fig. 6e-g). The calculated piezoresponse hysteresis loops show that with the strain varying from tensile to compressive the maximum piezoresponse gradually decreases (Supplementary Table 2).

Supplementary Note 5. Structure and ferroelectric switching of $Y_{\alpha}FeO_{1.5(\alpha+1)}$ thin films.

The Y/Fe ratio of the "YIG"-composition film grown using the $Y_3Fe_5O_{12}$ target was analyzed by high-resolution XPS. Calibrated with the stoichiometric $YFeO_3$ target, the ratio of the integrated areas of Y 3d and Fe 2p core level spectra (Supplementary Fig. 7a) indicates a Y/Fe ratio of 0.60, or $Y_{0.75}Fe_{1.25}O_3$. Unexpectedly, the as-grown $Y_{0.75}Fe_{1.25}O_3$ film on NSTO substrate exhibits a perovskite structure (Supplementary Fig. 7b). To further study the morphology and composition of this Y-deficient sample, cross-sectional STEM imaging and EDS mapping were performed. This shows compositional heterogeneity (standard deviation of 2.67 at%) with regions having

excessive Y or Fe respectively (Supplementary Fig. 7d). Despite the heterogeneity in composition, both the relatively Y-poor and Y-rich regions exhibit a perovskite structure (Supplementary Fig. 7e).

To further characterize the structure, a high-resolution reciprocal space map around the 013 family of peaks was collected. As shown in Supplementary Fig. 7c, besides the 013 peak of NSTO substrate, only one broad film peak is present, indicating no apparent structural phase separation.

The codeposited $Y_{\alpha}FeO_{1.5(\alpha+1)}$ thin films all adopt the perovskite structure (Supplementary Fig. 7b), but the lattice parameter varies with α, indicated by the slight shift of film peaks. In other words, the lattice will gradually expand upon the incorporation of defects in both the Y-rich and the Y-deficient cases.

The piezoresponse hysteresis loops of samples with α = 1.11 and α = 1.03 are shown in Supplementary Fig. 7g, h. Plotting the 2 × remanent piezoresponse as a function of Y/Fe ratio (α) in Supplementary Fig. 7i, the piezoresponse increases as the sample becomes more Y-rich.

Supplementary Note 6. Defect stability calculations.

To estimate the relative stability of intrinsic point defects in $YFeO_3$, we first constructed the phase diagram (Supplementary Fig. 8a) and determined the range of chemical potentials (μ_{Fe}, μ_Y, μ_O) that stabilize $YFeO_3$ over Y_2O_3 and Fe_2O_3 oxides. The $YFeO_3$ is stable in the narrow region between the two parallel lines, with excess Y at the upper boundary with Y_2O_3 and excess Fe at the lower boundary with Fe_2O_3. We then chose the chemical potentials that correspond to O-rich/Y-rich experimental conditions (marked with a red star in Supplementary Fig. 8a) and calculated the defect stability in $YFeO_3$.

The defect formation energies were calculated using the following equation $E_{form}=E_{defect} - E_{perfect} + \sum n_i\mu_i$, where E_{defect} is the total energy of the supercell with the defect, $E_{perfect}$ is the total energy

of the perfect intrinsic supercell, μ_i represents the chemical potential of the corresponding atom n_i. All point defects were considered in their neutral states. An oxygen vacancy in an equatorial site was used to calculate defect formation energy. We employed PBE+U (U_{eff} = 4 eV for *d*- electrons of Fe) exchange-correlation functional taking into account corrections for O_2 overbinding and fitted chemical-potential corrections for metal ions [7]. A pseudocubic ($\sqrt{2}a_o$ x $\sqrt{2}\ b_o$ x c_o) supercell with a single defect and fixed in-plane lattice parameters was used to calculate defect formation energies and investigate the epitaxial strain effects.

Our thermodynamic analysis (Supplementary Fig. 8b) identified Y_{Fe} as the most energetically stable intrinsic defects under these chemical potential conditions in a good agreement with our experiments and with previous first-principles calculations of defects in yttrium iron garnet [8]. We also demonstrated that Y_{Fe} antisite defects become more stable under tensile epitaxial strain (e.g., induced by $SrTiO_3$ substrate), while compressive strain stabilizes Fe_Y antisite defects (Supplementary Fig. 8c). We note that the antisite defects have small but positive formation energies. This indicates that the YFO structure is stable, but the antisites are readily formed. Cation and anion vacancies have much greater formation energies.

We also evaluated the possibility of migration of Y_{Fe} antisite defect along the $[001]_o$ axis in an epitaxially strained supercell of $YFeO_3$. Since the migration of cations in most perovskites follows a vacancy-mediated mechanism[9,10], we introduced a Fe cation vacancy in the vicinity of the Y_{Fe} defect (see Supplementary Fig. 9c). The climbing image nudged elastic band (CI-NEB)[11] calculations revealed a high barrier of 3.1 eV (Supplementary Fig. 9d), indicating Y_{Fe} migration will not occur under the voltages used in this work.

Supplementary Note 7. Ferroelectric and magnetic properties calculations.

The calculated electronic properties show that Y-rich YFO (α=1.28) remains insulating even at high antisite defect concentration (Supplementary Fig. 9a). We then compared the relative stability of different $YFeO_3$ phases: centrosymmetric *Pbnm* and two ferroelectric phases (*Pna21* and *R3c*) under different epitaxial strain and Y/Fe stoichiometry. The *Pna21* phase was found to be unstable for all considered conditions, while *R3c* symmetry can be stabilized by a large epitaxial strain and Y/Fe non-ideal stoichiometry as seen in Supplementary Fig. 9b. Although this phase remains metastable under experimental conditions (*e.g.* grown on DSO, a =3.944 Å and STO, a =3.905 Å) substrates), the presence of Y_{Fe} antisite defects could stabilize *R3c*-like regions within the *Pbnm* structure. Note that the epitaxial strain is essential for the stabilization of *R3c*-like regions which are not observed in fully relaxed supercells.

The choice of exchange-correlation functional is known to greatly affect spontaneous polarization and switching barriers in ferroelectric materials [12]. We investigated the effect of various exchange-correlation functional and their combination with a small Coulomb U on ferroelectric polarization and switching barrier of Y-rich $YFeO_3$.

Our calculations revealed that ferroelectric properties of Y-rich $YFeO_3$ decrease significantly when DFT+U with large effective Coulomb U (U_{eff} =4 eV) are employed (Supplementary Table 3). The spontaneous polarization of Y-rich $YFeO_3$ calculated using LDA+U (U_{eff}=1 eV), PBE, PBEsol and PBEsol+U (U_{eff} =1 eV) and HSE functional lies in the range of 3.2-7.2 μC/cm^2, in a reasonable agreement with experimental results.

Our calculations also indicate that the polarizations of neighboring Y_{Fe} do not cancel out each other and almost equal polarization amplitude is observed for both ordered and arbitrarily distributed Y_{Fe} antisites at Y/Fe = 1.13 (Supplementary Fig. 10).

The switching barrier calculated with the discussed above methods are underestimated with the largest activation energy of 57 meV calculated using LDA+U (U_{eff} =1 eV) method. The latter approach is chosen to calculate ferroelectric properties of other orthoferrites (Fig. 4d in the main text). HSE hybrid functional was used to reduce a self-interaction error in the electronic structure calculations.

The magnetic configuration was also modeled using DFT. Orthoferrites in general are orthorhombic perovskites in which the primary magnetic order is a G_x-type antiferromagnet configuration of Fe, *i.e.,* nearest neighbor Fe^{3+} have opposite magnetic moments, and these moments point along x. Canting leads to a $G_xA_yF_z$ spin structure with the F_z term denoting a small ferromagnetic moment along the orthorhombic c-axis. Taking the relaxed structure, either pristine (stoichiometric) or with an antisite defect, we compared the stability of the G-type AFM with ferromagnetic (FM) configurations. The total energy (eV/f.u.) is given in Supplementary Table 4. AFM order is significantly more stable for both structures. We also checked the local order by switching one of the Fe moments adjacent to the Y_{Fe} defect. The calculation converged back to the G-type state, *i.e.,* none of the neighbors of the antisite defect was stable with FM order. Therefore, the AFM configuration is robust to the perturbation introduced by the antisite Y_{Fe}. A single antisite in the unit cell yields a net magnetization of ~5μ_B, equal to the moment of one Fe^{3+}, as expected. However, when averaged over a random distribution of antisites, which are present on both Fe sublattices, these moments compensate and the material remains antiferromagnetic.

Supplementary Table 1 | XPS Composition analysis of five YFO films prepared from different PLD runs under the same conditions on NSTO substrates.

	High-resolution XPS					
Sample	#1	#2	#3	#4	#5	Average
Y/Fe	1.19	1.22	1.20	1.23	1.13	1.19±0.04

Supplementary Table 2 | Lattice parameters of YFO films grown on different substrates

Sample	Substrate lattice parameter (Å)	YFO film				
		lattice parameters (Å)		unit cell volume (Å^3)	Y/Fe ratio	2 × remnant piezoresponse (pm)
		a_p	c_p			
YFO/SRO/DSO	3.944	3.862	3.812	56.83	1.12	18.5
YFO/SRO/STO	3.905	3.861	3.817	56.90	1.11	20.2
YFO/LSMO/LSAT	3.868	3.856	3.829	56.93	1.19	5.61
YFO/LSMO/LAO	3.788	3.789*	3.865	55.49	1.17	3.81
Bulk YFO	*N/A*	*3.847*	*3.803*	*56.28*	*1.00*	*N/A*

* This value is estimated assuming the YFO thin film is fully strained to the substrate. A strain relaxation due to the large lattice mismatch would lead to a larger in-plane lattice parameter and unit cell volume.

Supplementary Table 3 | Ferroelectric properties of Y-rich $YFeO_3$ using LDA, PBE, PBEsol functional and their combination with Coulomb U.

Functional	Polarization, μC/cm^2	Switching barrier, meV
PBE	6.6	43
LDA	metallic	-
PBEsol	6.2	27
PBEsol+U1	4.2	21
PBEsol+U4	0.9	4
LDA+U1	7.2	57
LDA+U4	1.3	5
HSE	3.2	18

* Calculations are carried out for $\sqrt{2}a_p$ x $\sqrt{2}b_p$ x c_p *Pbnm* supercell assuming epitaxial growth on STO substrate. Ferroelectric polarization along out-of-plane (c_p) direction is calculated using Berry Phase approach. Switching barriers are calculated using CI-NEB approach.

Supplementary Table 4 | Total energy (eV/f. u.) for $YFeO_3$ with FM or AFM magnetic order

Magnetic order	Pristine (no antisite)	With Y_{Fe} antisite (Y/Fe = 1.28)
FM	-39.95	-40.54
AFM	-40.16	-40.68

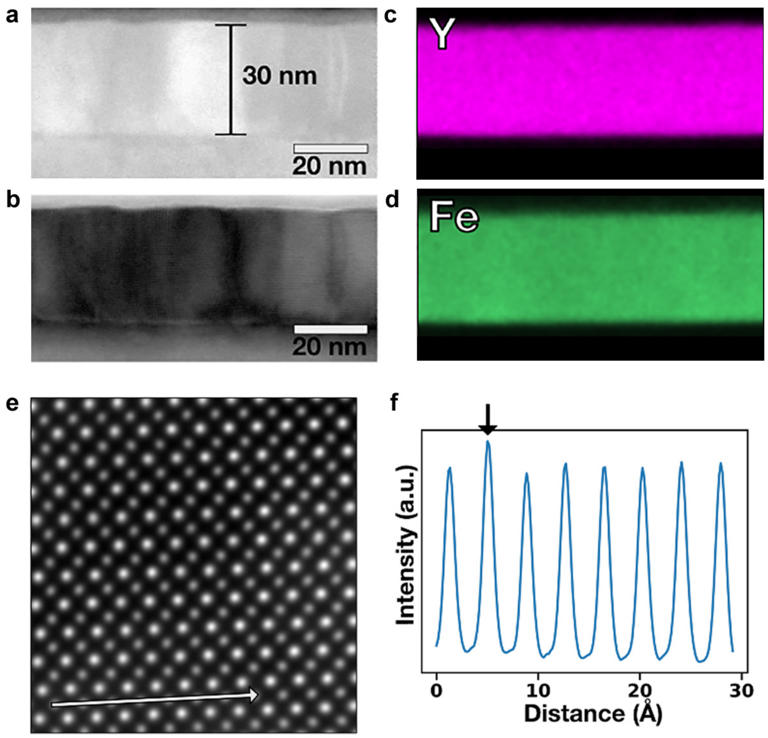

Supplementary Fig. 1 | STEM of the YFO/NSTO sample. a-d, Cross-sectional STEM image of high-angle annular dark-field (HAADF) (**a**) and bright field (BF) (**b**) and EDS mapping of Y (**c**) and Fe (**d**) collected from a YFO thin film grown on a NSTO substrate in cross section along the pseudocubic [100]. BF STEM image reveals that the YFO thin film shows mosaicity with slight deviations of the crystal orientations. **e**, High-resolution HAADF STEM image of the film. White line = 28Å. **f**, HAADF STEM intensity profile along the line shown in (**e**). Note the Fe-O atom column with increased intensity (arrow) contains Y_{Fe} defects.

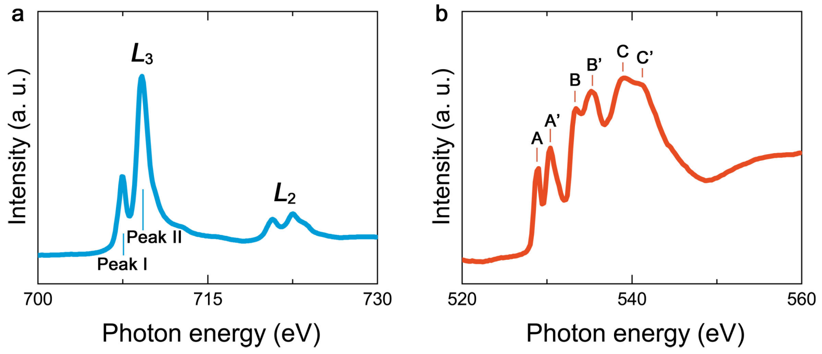

Supplementary Fig. 2 | XAS analysis of as-prepared YFO thin films. XAS spectra at Fe *L*-edge (**a**) and O *K*-edge (**b**).

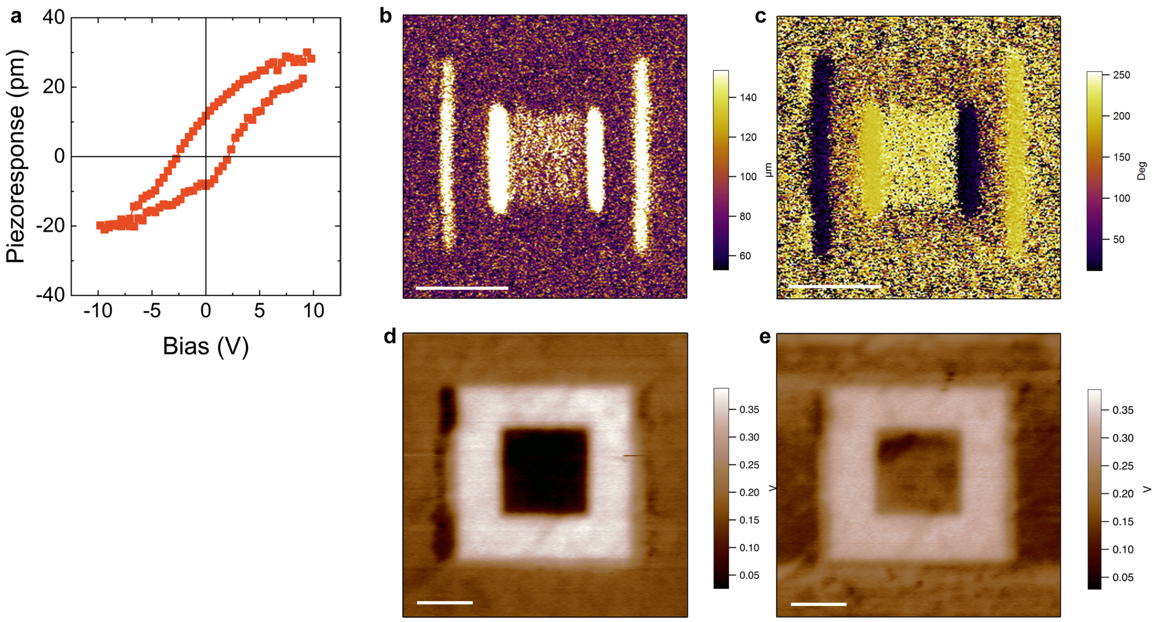

Supplementary Fig 3 | Ferroelectric properties of YFO/NSTO. a, Piezoresponse hysteresis loop calculated with the amplitude and phase data. **b**, **c**, Lateral PFM amplitude (**b**) and phase (**c**) images. **d**, **e**, SKPFM images taken right after the poling (**d**) and after multiple contact-mode scans with tip grounded (**e**), respectively. The scale bars in (**b-e**) all correspond to 1μm.

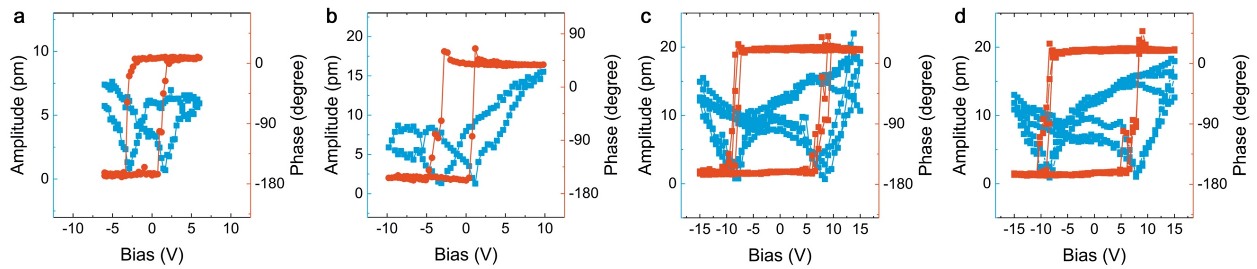

Supplementary Fig. 4 | SS-PFM of YFO/NSTO with varying thicknesses and temperature. SS-PFM amplitude curves and phase loops of the YFO thin films with thickness of 10 nm (**a**) and 100 nm (**b**) measured at room temperature, and of the 30-nm-thick YFO films measured at elevated temperatures, *i.e.* 100 °C (**c**) and 150 °C (**d**), respectively.

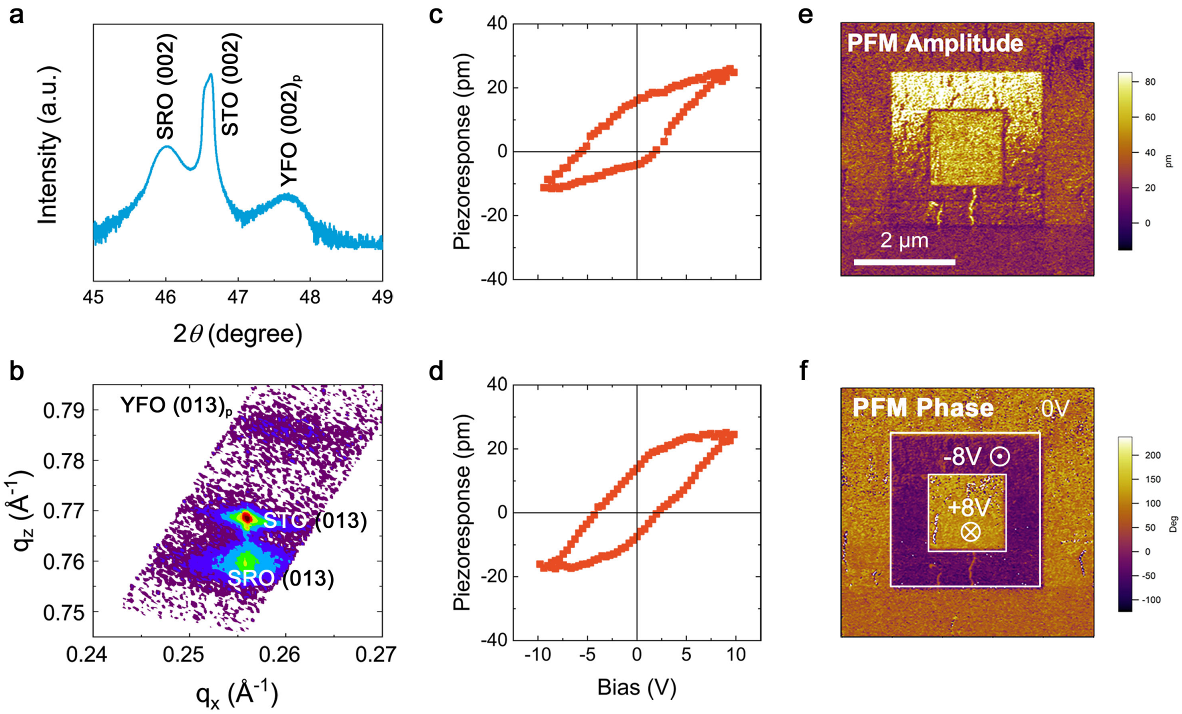

Supplementary Fig. 5 | Structural and PFM characterization on YFO/SRO/STO. **a**, **b**, Structure characterization of YFO/SRO/STO by XRD (**a**) and RSM (**b**). **c**, **d**, Calculated piezoresponse hysteresis loops with the cantilever loaded directly on the surface of YFO film (**c**) and on the top of Pt electrode (**d**). **e**, **f**, Vertical PFM amplitude (**e**), and vertical PFM phase (**f**) images collected after writing with voltages of +/−8 V applied on the tip.

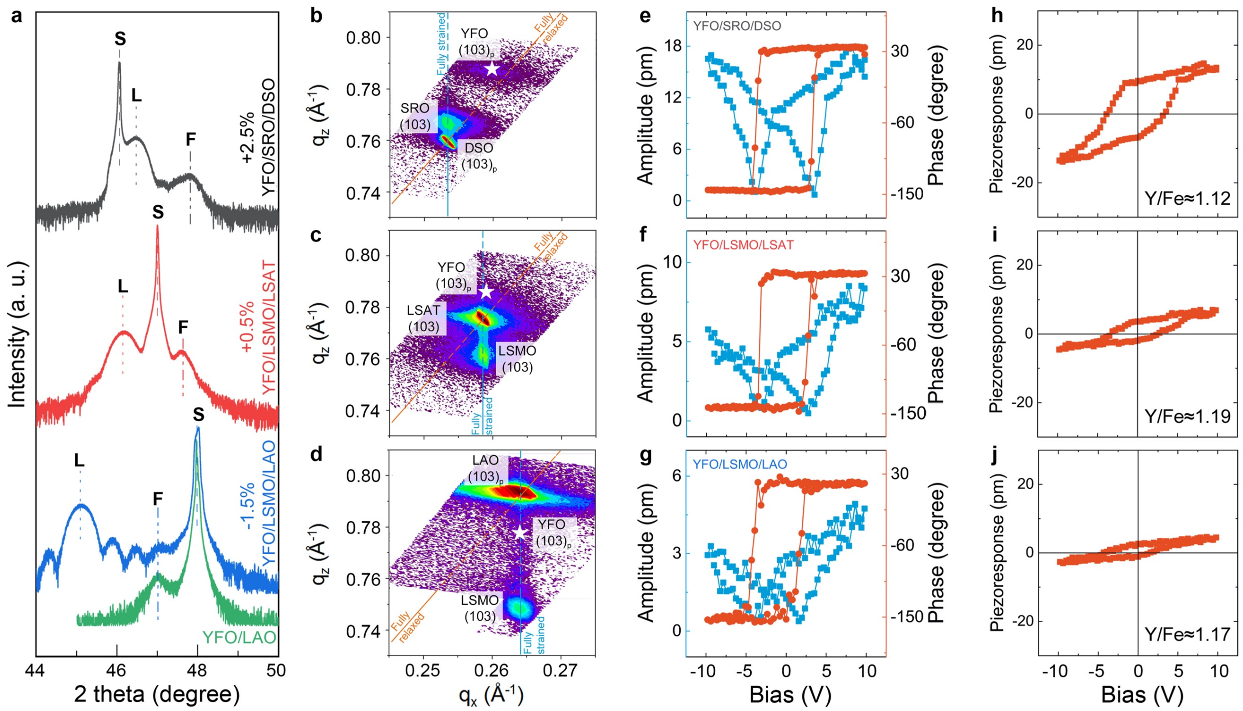

Supplementary Fig. 6 | Structure and ferroelectric switching of YFO films on different substrates. **a**, High-resolution XRD pattern of the 002 family of peaks of YFO thin films grown on SRO/DSO, LSMO/LSAT, LSMO/LAO, and bare LAO substrates. The **S**, **L** and **F** refer to the peaks of substrate, conductive layer, and YFO thin film, respectively. **b-d**, 013 RSM of YFO films grown on SRO/DSO (**b**), LSMO/LSAT (**c**) and LSMO/LAO (**d**), respectively. The blue and orange dash lines indicate fully strained and fully relaxed states, respectively. The film peaks of those grown on LSMO/LSAT and LSMO/LAO are partially overlapped with the substrate signals. **e-g**, Local vertical SS-PFM amplitude curves and phase loops of the films grown on SRO/DSO (**e**), LSMO/LSAT (**f**) and LSMO/LAO (**g**), respectively. **h-j**, Piezoresponse hysteresis loops of films grown on SRO/DSO (**h**), LSMO/LSAT (**i**) and LSMO/LAO (**j**), respectively.

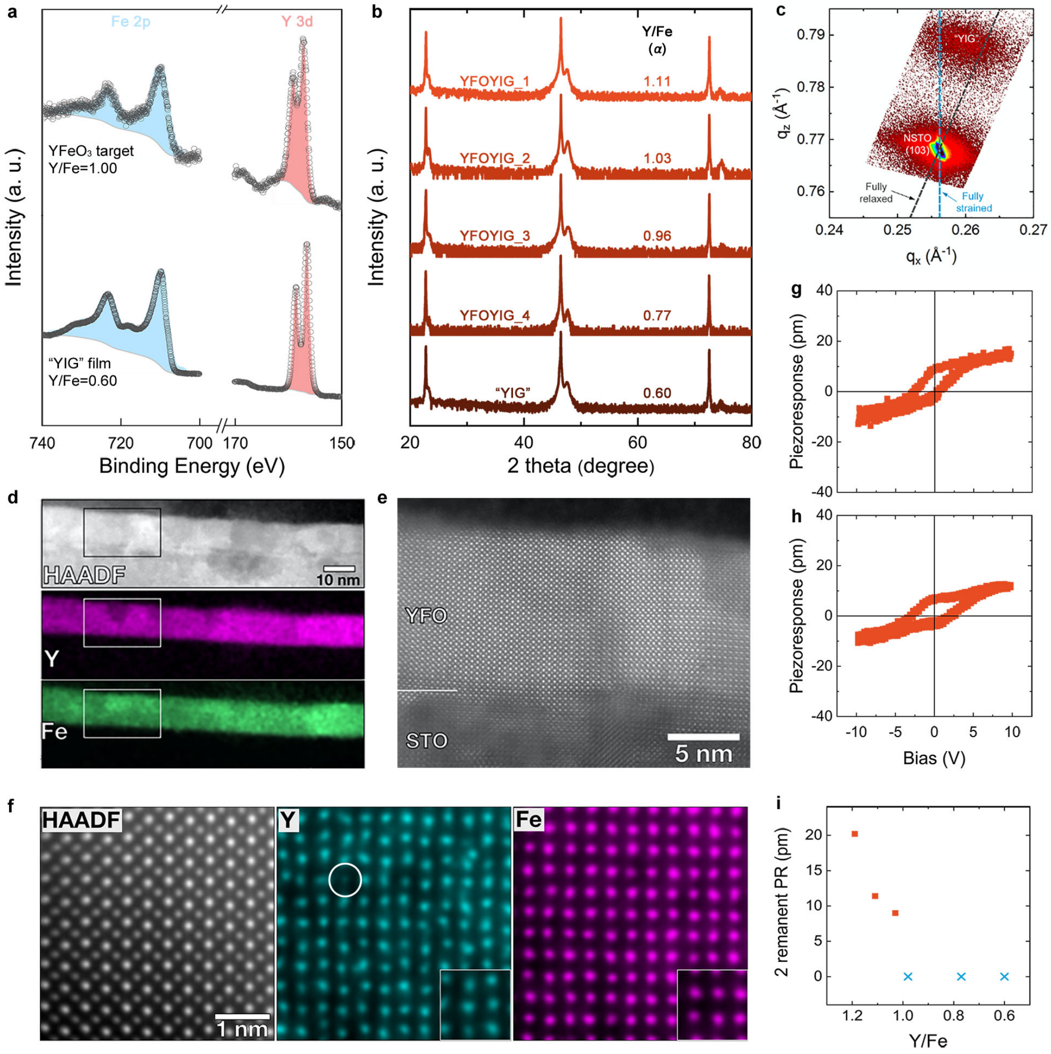

Supplementary Fig. 7 | "YIG" ($Y_{0.75}Fe_{1.25}O_3$) and $Y_{\alpha}FeO_{1.5(\alpha+1)}$ thin films. **a**, High-resolution XPS of Fe 2p and Y 3d core level spectra of the stoichiometric $YFeO_3$ target and the "YIG" $Y_{0.75}Fe_{1.25}O_3$ film on NSTO substrate. **b**, XRD patterns of the $Y_{0.75}Fe_{1.25}O_3$ film and codeposited $Y_{\alpha}FeO_{1.5(\alpha+1)}$ thin films. **c**, 013 RSM of the $Y_{0.75}Fe_{1.25}O_3$ film. **d**, HAADF STEM image and EDS maps of $Y_{0.75}Fe_{1.25}O_3$ film on NSTO substrate. **e**, Enlarged HAADF STEM image including both Y-poor and Y-rich regions as indicated by the rectangular in (**d**). **f**, Atomic-resolution STEM EDS elemental mapping on the Y-poor region. The circle reveals a signature of Y deficiency due to its much weaker X-ray intensity. **g**, **h**, Calculated piezoresponse hysteresis loops of samples with α = 1.11 (**g**) and α = 1.03 (**h**). **i**, 2 × remnant piezoresponse as a function of Y/Fe ratio (α).

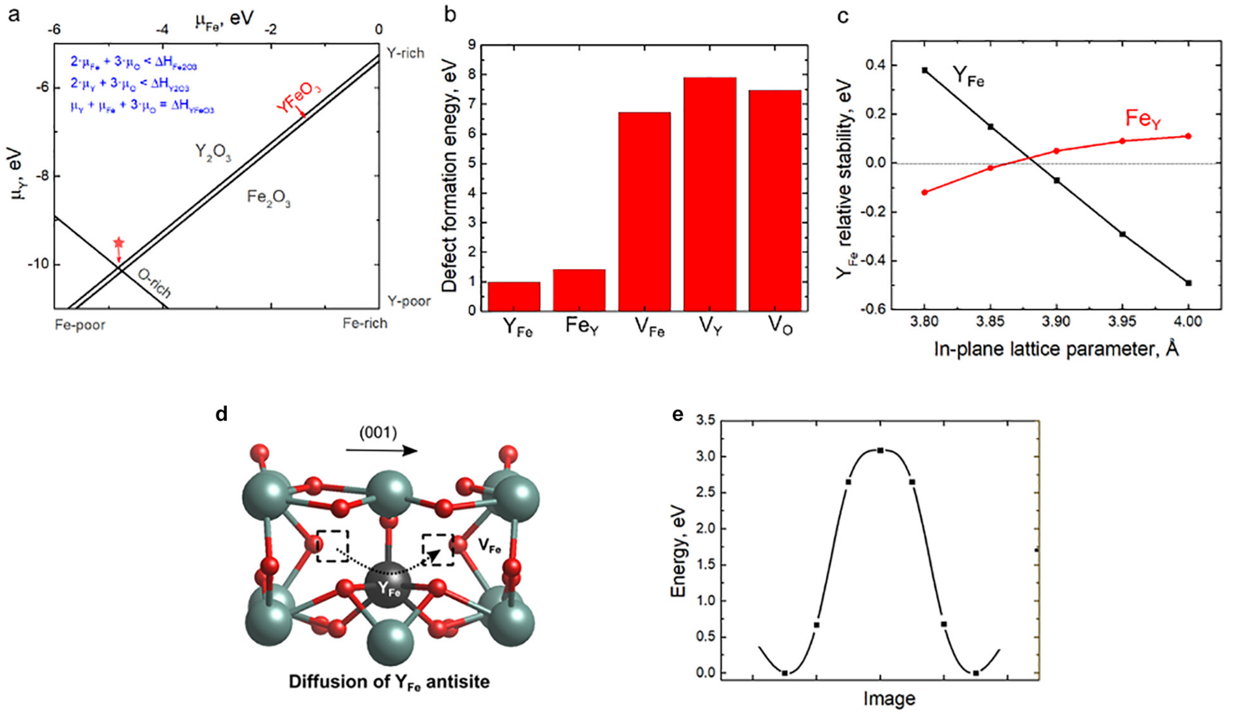

Supplementary Fig. 8 | Evaluation of defect stability and migration. a, the phase diagram of $YFeO_3$. The region of Y, Fe and O chemical potentials with which $YFeO_3$ is stable is indicated by the red arrow. Red star indicates the conditions used to calculate defect formation energies. **b**, the defect formation energies per $\sqrt{2}\,a_o$ x $\sqrt{2}\,b_o$ x 2 c_o supercell of $YFeO_3$. In-plane lattice parameters were fixed to 3.90 Å assuming epitaxial growth on Nb-doped $SrTiO_3$ substrate. **c**, the relative stability of Y_{Fe} and Fe_Y antisite defects in $\sqrt{2}\,a_o$ x $\sqrt{2}\,b_o$ x 2 c_o supercell for different in-plane lattice parameters. Defect stability in the fully relaxed orthorhombic supercell was used as a reference. **d**, **e**, the activation barrier of Y_{Fe} diffusion via Fe vacancy (V_{Fe}) mediated mechanism in $\sqrt{2}\,a_o$ x $\sqrt{2}\,b_o$ x 2 c_o supercell of $YFeO_3$. Snapshot represents Y_{Fe} position in the saddle point on a potential energy surface.

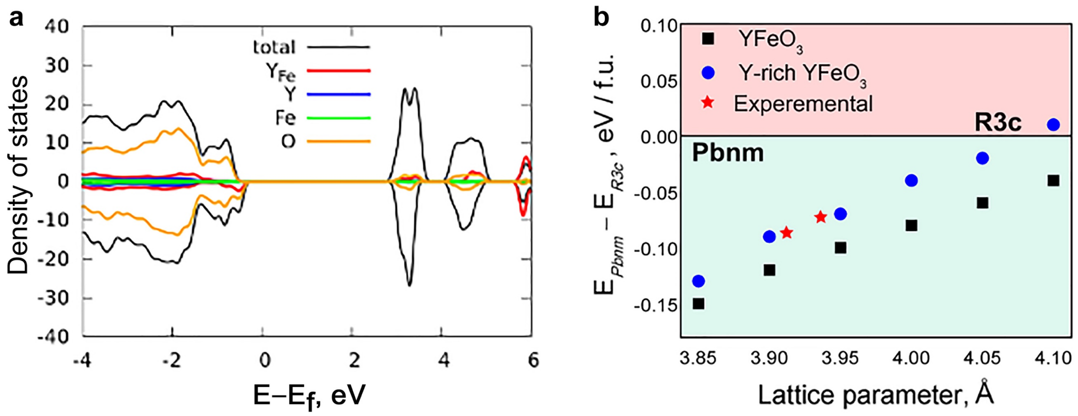

Supplementary Fig. 9 | DFT calculations. **a**, projected density of states of Y-rich YFO (α = 1.28). Fermi level is set to zero. **b**, relative stability of *Pbnm* and *R3c* phases of YFO and Y-rich YFO (α = 1.28). Stars indicate substrate lattice parameters considered in our experiment.

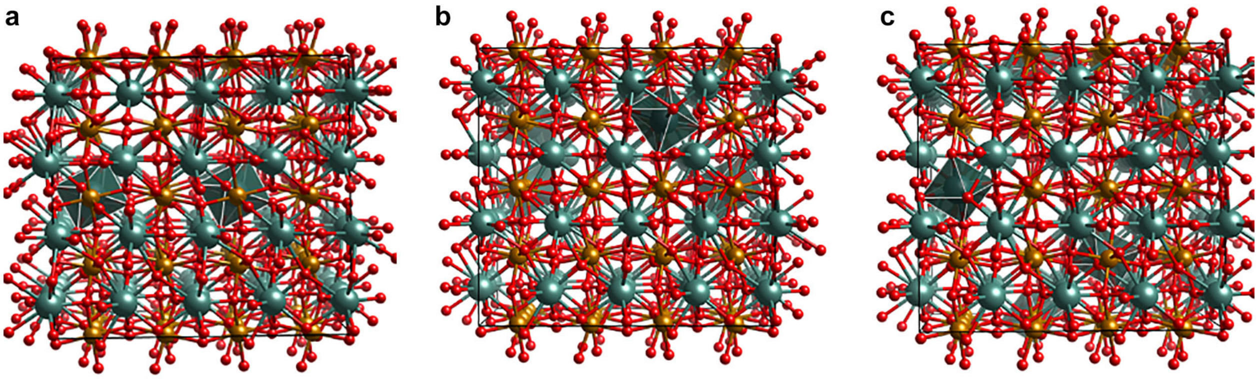

Supplementary Fig. 10 | Calculated ferroelectric polarization for three different Y_{Fe} defect distributions (Y/Fe = 1.13) in the YFO epitaxially grown on $SrTiO_3$ substrate. Y, Fe and O atoms are in green, brown and red. Y_{Fe} positions are highlighted with polyhedra. The calculated polarization along (001) direction is 3.4, 2.9 and 3.6 μC/cm^2 for YFO with ordered (a), cluster-like (b) and arbitrarily distributed (c) Y_{Fe} defects, respectively.

References

1. Hayes, J. R. & Grosvenor, A. P. An x-ray absorption spectroscopic study of the electronic structure and bonding of rare-earth orthoferrites. *J. Phys. Condens. Matter.* **23**, 465502 (2011).
2. Qi, X., Dho, J., Tomov, R., Blamire, M. G. & MacManus-Driscoll, J. L. Greatly reduced leakage current and conduction mechanism in aliovalent-ion-doped $BiFeO_3$. *Appl. Phys. Lett.* **86**, 062903 (2005).
3. Pabst, G. W., Martin, L. W., Chu, Y.-H. & Ramesh, R. Leakage mechanisms in $BiFeO_3$ thin films. *Appl. Phys. Lett.* **90**, 072902 (2007).
4. Zhou, Z. et al. Ferroelectric domains and phase transition of sol-gel processed epitaxial Sm-doped $BiFeO_3$ (001) thin films. *J. Materiomics* **4**, 27-34 (2018).
5. Zayak, A. T., Huang, X., Neaton, J. B. & Rabe, K. M. Structural, electronic, and magnetic properties of $SrRuO_3$ under epitaxial strain. *Phys. Rev. B* **74**, 094104 (2006).
6. Seo, H., Posadas, A. & Demkov, A. A. Strain-driven spin-state transition and superexchange interaction in $LaCoO_3$: Abinitiostudy. *Phys. Rev. B* **86**, 014430 (2012).
7. Kirklin, S. et al. The Open Quantum Materials Database (OQMD): assessing the accuracy of DFT formation energies. *npj Comput. Mater.* **1**, 15010 (2015).
8. Tan, S., Zhang, W., Yang, L., Chen, J. & Wang, Z. Intrinsic defects in yttrium iron garnet: A first-principles study. *J. Appl. Phys.* **128**, 183904 (2020).
9. Walsh, A., Catlow, C. R. A., Smith, A. G. H., Sokol, A. A. & Woodley, S. M. Strontium migration assisted by oxygen vacancies in $SrTiO_3$ from classical and quantum mechanical simulations. *Phys. Rev. B* **83**, 220301 (2011).
10. Uberuaga, B. P. & Vernon, L. J. Interstitial and vacancy mediated transport mechanisms in perovskites: A comparison of chemistry and potentials. *Solid State Ionics* **253**, 18-26 (2013).
11. Henkelman, G., Uberuaga, B. P. & Jonsson, H. A climbing image nudged elastic band method for finding saddle points and minimum energy paths. *J. Chem. Phys.* **113**, 9901-9904 (2000).
12. Zhang, Y., Sun, J., Perdew, J. P. & Wu, X. Comparative first-principles studies of prototypical ferroelectric materials by LDA, GGA, and SCAN meta-GGA. *Phys. Rev. B* **96**, 035143 (2017).
